# Insights into 2D MXenes for Versatile Biomedical Applications: Current Advances and Challenges Ahead

**DOI:** 10.1002/advs.201800518

**Published:** 2018-07-19

**Authors:** Han Lin, Yu Chen, Jianlin Shi

**Affiliations:** ^1^ State Key Laboratory of High Performance Ceramics and Superfine Microstructures Shanghai Institute of Ceramics Chinese Academy of Sciences Shanghai 200050 P. R. China; ^2^ University of Chinese Academy of Sciences Beijing 100049 P. R. China

**Keywords:** materials science, MXenes, nanomedicines, surface chemistry, theranostics

## Abstract

Great and interdisciplinary research efforts have been devoted to the biomedical applications of 2D materials because of their unique planar structure and prominent physiochemical properties. Generally, ceramic‐based biomaterials, fabricated by high‐temperature solid‐phase reactions, are preferred as bone scaffolds in hard tissue engineering because of their controllable biocompatibility and satisfactory mechanical property, but their potential biomedical applications in disease theranostics are paid much less attention, mainly due to their lack of related material functionalities for possibly entering and circulating within the vascular system. The emerging 2D MXenes, a family of ultrathin atomic nanosheet materials derived from MAX phase ceramics, are currently booming as novel inorganic nanosystems for biologic and biomedical applications. The metallic conductivity, hydrophilic nature, and other unique physiochemical performances make it possible for the 2D MXenes to meet the strict requirements of biomedicine. This work introduces the very recent progress and novel paradigms of 2D MXenes for state‐of‐the‐art biomedical applications, focusing on the design/synthesis strategies, therapeutic modalities, diagnostic imaging, biosensing, antimicrobial, and biosafety issues. It is highly expected that the elaborately engineered ultrathin MXenes nanosheets will become one of the most attractive biocompatible inorganic nanoplatforms for multiple and extensive biomedical applications to profit the clinical translation of nanomedicine.

## 1 Introduction

The rapid progress of clinic biomedicine and nanobiotechnology has stimulated the generation of diverse novel inorganic nanosystems, which offers multiple theranostic modalities as potential alternatives in combating various diseases by synergistic therapy and multimodal imaging, especially in cancer theranostics.^1^, ^2^, ^3^, ^4^, ^5^, ^6^, ^7^, ^8^, ^9^, ^10^, ^11^ Currently, great efforts for multidisciplinary research have been focused on biomedical applications of 2D nanomaterials, a newly emerging subtype of nanomaterials with ultrathin layer‐structured topology, including mostly explored graphene and its derivatives,^12^, ^13^ hexagonal boron nitrides (h‐BN),^14^ transition metal dichalcogenides (TMDCs),^15^ transition metal oxides (TMOs),^16^ palladium (Pd) nanosheets,^17^ black phosphorus (BP),^18^, ^19^ and transition metal carbides (MXenes).^20^ Their multifaceted properties, such as high specific surface area and intriguing physiochemical natures, make them able to satisfy the strict demands in theranostic nanomedicine such as drug delivery, phototherapy, diagnostic imaging, biosensing, and even tissue engineering.^21^, ^22^, ^23^, ^24^, ^25^, ^26^, ^27^, ^28^, ^29^

MXenes, a new family of multifunctional 2D solid crystals containing a large class of transition metal carbides, nitrides, and carbonitrides with metallic conductivity and hydrophilic nature, as well as excellent mechanical properties, were developed by Barsoum and co‐workers.^20^, ^30^, ^31^, ^32^ M*_n_*_+1_X*_n_* layer (named as MXene) was fabricated by the selective extraction of A‐element from layered ternary carbides of M*_n_*_+1_AX*_n_* phases (*n* = 1–3), where M is an early transition metal, A is an A group element, and X is C or N.^33^ MXenes typically have three different formulas: M_2_X, M_3_X_2_, and M_4_X_3_. The versatile chemistry of MXenes has found numerous applications in energy storage,^34^, ^35^, ^36^, ^37^, ^38^, ^39^ water purification,^40^ chemical sensors, photo‐ or electro catalysis,^41^ and electromagnetic interference shielding.^42^, ^43^ They also hold great potentials in the biomedical field. On one hand, the high specific surface areas enable the MXene nanosheets to be potential drug or protein carriers with abundant anchoring sites and reservoirs. The ultrathin layered structure with almost single‐atomic thickness endows MXenes with fascinating physiochemical properties (e.g., photothermal conversion,^44^, ^45^ electron transparency, X‐ray attenuation,^46^, ^47^ and localized surface plasmon resonance^48^) and biological behaviors (e.g., enzyme‐triggered biodegradation,^49^ cellular endocytosis,^50^ distinct biodistribution, and metabolism pathway^49^). On the other hand, the controllable component and tunable in‐plane structure of MXenes can be precisely designed and synthesized in the pristine structure of MAX phases, creating flexible/extensive multifunctionalities of MXenes in promising theranostic nanomedicine. To date, the MXenes with various attractive physicochemical properties and biological effects, and the cutting‐edge researches for emerging 2D materials, have attracted increasing attention in scientific community of nanomedicine.

**Figure 1:**
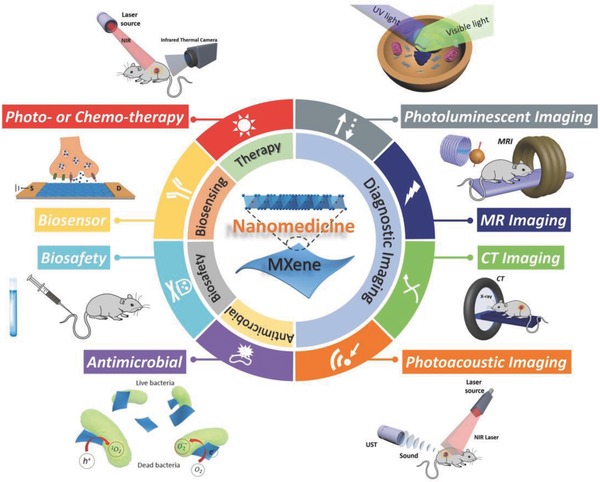
Summary of emerging 2D MXenes used in nanomedicine. Summative scheme of emerging 2D MXenes for biomedical applications, and schematic illustration of the 2D MXene‐based nanomedical applications, including therapeutic practice, diagnostic imaging, biosensing, antimicrobial, and biosafety evaluations.

In this review, we summarize and discuss the current state‐of‐the‐art of 2D MXenes as a robust nanoplatform on the basis of synthetic methods, surface chemistry, and biomedical applications, as well as the related challenges and perspectives for future developments (**Figure** **1**). To be specific, the derivatives of emerging research of 2D MXenes in nanomedicine could be categorized into therapeutic modality,^45^, ^46^, ^47^, ^48^, ^49^, ^51^, ^52^ diagnosis imaging,^46^, ^47^, ^49^, ^51^, ^53^ biosensing,^54^, ^55^, ^56^, ^57^ antimicrobial,^58^, ^59^ and biosafety evaluation.^50^ The bigger picture is that by gaining deeper insights into the material science and biological behavior of 2D MXene nanosheets for existing and emerging biomedical modalities, we will be able to facilitate immense and promising applications with clinical‐translation potential in benefitting the human health.

## 2 Synthetic Methods and Surface Chemistry

The synthetic methodologies of 2D layered nanomaterials can be divided into two distinct routes: (i) top‐down approach, and (ii) the bottom‐up method.^60^, ^61^, ^62^, ^63^ Both strategies have been performed on the fabrication of single‐, few‐layer, or multilayer nanostructure of MXenes.

### 2.1 Top‐Down Synthesis

**Figure 2:**
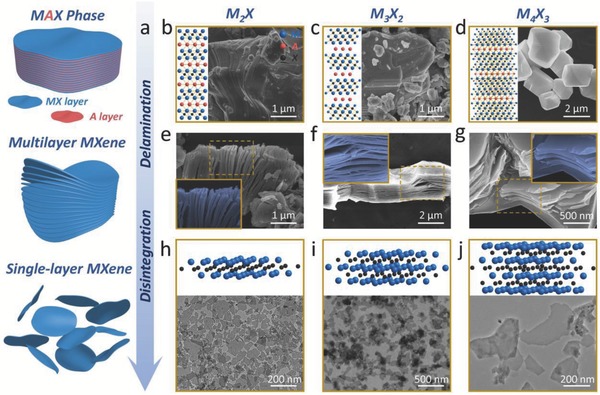
Synthetic methods of MXenes for biomedicine. a) Schematic diagram for the synthesis of biocompatible MXenes, including HF etching (delamination), organic base molecules intercalation (disintegration), and surface functionalization with organic molecules or inorganic nanoparticles (surface modification). b) 2D ball‐and‐stick models and SEM images of precursor MAX phase for M_2_X, e) SEM image of multilayer M_2_X, and h) 3D ball‐and‐stick models and TEM image single‐layer M_2_X‐based MXenes. Reproduced with permission.^49^ Copyright 2017, American Chemical Society. c) 2D ball‐and‐stick models and SEM images of precursor MAX phase for M_3_X_2_, f) SEM image of multilayer M_3_X_2_, and i) 3D ball‐and‐stick models and TEM image single‐layer M_3_X_2_‐based MXenes. Reproduced with permission.^45^ Copyright 2017, American Chemical Society. d) 2D ball‐and‐stick models and SEM images of precursor MAX phase for M_4_X_3_, g) SEM image of multilayer M_4_X_3_, and j) 3D ball‐and‐stick models and TEM image single‐layer M_4_X_3_‐based MXenes. Reproduced with permission.^47^ Copyright 2018, Wiley‐VCH.

The top‐down method is based on the direct exfoliation of bulk crystals, which employs various driving forces including mechanical and chemical exfoliations. To date, the general focus of MXenes' fabrication is on liquid‐phase exfoliation, a facile and high‐yield process, which has been proven to be of high efficiency in the production of ultrathin, nanoscale MXenes (**Figure** **2**a). In brief, the transformation from parent MAX‐phase ceramics (Figure 2b–d) to nanoscale 2D MXenes undergoes the following two steps: delamination by hydrofluoric acid (HF) etching to obtain the multilayer‐stacked MXenes (Figure 2e–g), and disintegration by organic base molecules intercalation or probe sonication breakage to acquire few‐layer or single‐layer MXenes (Figure 2h–j). Benefiting from this methodology, nearly all types of MXenes could be obtained with diversified morphologies of few‐ or single‐layered nanosheets, featuring nanoscale‐lateral size and atomic‐scale thickness.

Since the first MXene, multilayered Ti_3_C_2_, was developed in 2011, a series of 2D MXenes such as multilayered Mo_2_C, V_2_C, Nb_2_C, Zr_3_C_2_, and Ta_4_C_3_, have been widely investigated and extensively explored of their performances and versatile applications.^30^, ^31^, ^64^, ^65^, ^66^, ^67^ These multilayered MXenes with large lateral size of sheets were produced by traditional synthesis via liquid exfoliation approach like HF etching, which might lead to poor therapeutic efficacy and potential biosafety issue. In particular, biomedical applications necessitate small‐enough lateral size and ultrathin thickness of these 2D nanoagents to facilitate their effective transport within the blood‐circulatory system, guarantee the high accumulation/penetration into lesion location, and enable the easy excretion out of the living body.^25^, ^27^, ^68^ Very recently, the typical multilayered Ti_3_C_2_, as well as the emerging MXene compounds of Nb_2_C and Ta_4_C_3_, has been successfully delaminated and disintegrated to produce single‐layer MXene with nanoscale‐lateral sizes (around 100 nm) and single‐atomic thickness (almost 1 nm), making the biomedical uses of MXene possible.^45^, ^49^

### 2.2 Bottom‐Up Synthesis

The bottom‐up approaches are the alternative method to produce 2D nanomaterials via atomic level control of their composition and morphology. It could be applied to fabricate 2D nanomaterials which were not easy to obtain though direct exfoliation from bulk. Generally, chemical vapor deposition (CVD) growth^69^, ^70^, ^71^, ^72^ and wet‐chemical synthesis^73^, ^74^, ^75^ are the major synthetic approaches based on the bottom‐up method to develop layered materials with high production quality. Compared to traditional family of 2D materials, the current advances of bottom‐up approach to synthesize MXene show yet to be successful, perhaps owing to the complex atoms and structures of MXenes' in‐layer morphology.

As a paradigm, Ren and co‐workers report the growth of 2D ultrathin α‐Mo_2_C crystals with large area and high quality via CVD in 2015.^76^ Utilizing methane as the carbon source, Cu/Mo foils used as the substrate could grow into 2D ultrathin α‐Mo_2_C crystals at temperatures above 1085 °C, which features the thickness of few nanometers and lateral size of over 100 µm. Such a high growth temperature guarantees the melting of metal Cu and the formation of Mo‐Cu alloy at the liquefied Cu/Mo interface. Mo atoms could then diffuse onto the surface of the liquid Mo‐Cu alloy to grow 2D Mo_2_C crystals by reacting with the decomposed methane. Importantly, such a CVD growth strategy developed toward transition metal carbides is facile and versatile, which offers a general strategy for synthesizing varied types of high‐quality 2D ultrathin transition metal carbides crystals. Moreover, other high‐quality 2D MXene‐structural crystals, such as WC and TaC crystals with a large lateral size and few defects, were also obtained by replacing the Mo foils with the W or Ta foils during the similar CVD process.^76^

### 2.3 Surface Modification and Multifunctionalization

MXenes reported so far commonly feature specific surface terminations including hydroxyl (—OH), oxygen (—O), or fluorine (—F), imparting hydrophilic nature to their surfaces, which, together with the high surface charge (negative zeta potential exceeding −40 mV), lead to high stability of surfactant‐free water‐based colloidal solutions.^20^, ^77^ However, suffering from rapid aggregation and precipitation in biological mediums similar to most nanomaterials in biomedicine, the delaminated ultrathin MXenes are usually unstable in complex physiological conditions and lack of multifunctionalization.^25^, ^27^ Thus, surface engineering is critical to endow these nanosystems with high stability and dispersity in a physiological environment, as well as multipurpose strategy by decoration of other functional materials.

**Figure 3:**
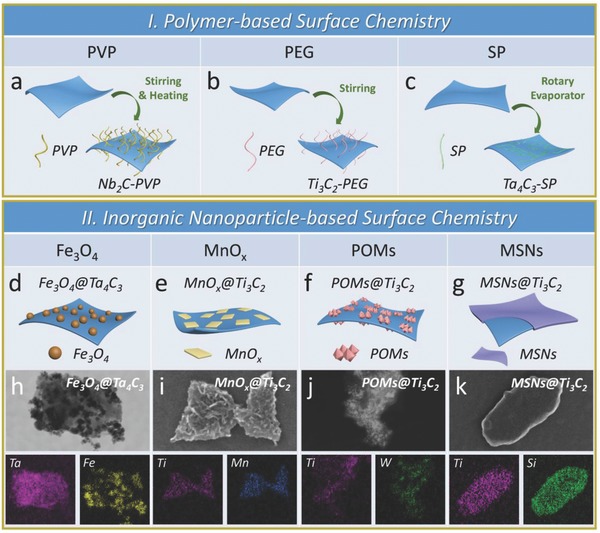
Surface chemistry of MXenes for biomedicine. Schematic illustrations of polymer‐based surface chemistry of MXenes. a) PVP modification of M_2_X (e.g., Nb_2_C nanosheets). b) PEGylation of M_3_X_2_ (e.g., Ti_3_C_2_ nanosheets). c) SP modification M_4_X_3_‐based MXenes. Schematic representations of inorganic nanoparticle‐based surface chemistry of MXenes. d) Superparamagnetic iron oxide (Fe_3_O_4_) nanoparticles grew onto the surface of Ta_4_C_3_ MXene by an in situ redox reaction. Reproduced with permission.^78^ Copyright 2018, Ivyspring International Publisher. e) In situ growth of small MnO*_x_* nanosheets on the surface of Ti_3_C_2_ according to a facile redox reaction. Reproduced with permission.^51^ Copyright 2017, American Chemical Society. f) Integration of GdW_10_‐based polyoxometalates (POMs) onto the surface of Ti_3_C_2_ MXene though an amide bond. Reproduced with permission.^79^ Copyright 2018, Springer. g) Surface nanopore engineering of silica (SiO_2_) on the 2D Ti_3_C_2_ MXene based on a process of sol‐gel chemistry. Reproduced with permission.^80^ Copyright 2018, Wiley‐VCH. The STEM images and the corresponding element mappings of h) Fe_3_O_4_@Ta_4_C_3_, i) MnO*_x_*@Ti_3_C_2_, j) POMs@Ti_3_C_2_, and k) SiO_2_@Ti_3_C_2_ composite MXenes.

In general, the surface modification and engineering of MXenes have been focused on the following two approaches: one is polymer‐based strategy of surface chemistry, modifying surface with certain molecules or polymers based on noncovalent interaction. For instance, the Nb_2_C nanosheets were decorated with polyvinylpyrrolidone (PVP) molecules (**Figure** **3**a).^49^ Moreover, the PEGylation in Ti_3_C_2_ nanosheets surface by electrostatic adsorption was achieved to maintain the stability of MXene in physiological conditions (Figure 3b),^48^ and the Ta_4_C_3_ nanosheets were successfully modified with soybean phospholipid (SP) (Figure 3c).^47^ The other is inorganic nanoparticle‐based surface chemistry, decorating MXenes with multifunctional inorganic nanoparticles which could further broaden their potential applications. For instance, the integration of Ta_4_C_3_ nanosheets with Fe_3_O_4_ nanoparticles (Figure 3d,h) and the combination of Ti_3_C_2_ and MnO*_x_* nanoparticles (Figure 3e,i) are typical paradigms of integrating therapeutic platforms with MRI contrast agents (CAs), endowed them with the capabilities of concurrent therapeutics and diagnostic imaging.^51^, ^78^ Similarly, the integration of 2D Ti_3_C_2_ MXene nanosheets with GdW_10_‐based polyoxometalates (POMs) provides phototherapeutic nanoplatform with magnetic resonance (MR) and/or computed tomography (CT) imaging guidance toward xenograft tumor (Figure 3f,j).^79^ Another typical paradigm is the integrating Ti_3_C_2_ MXene with the mesoporous silica nanoparticles (MSNs), a classic drug delivery system (DDS). In comparison to traditional 2D MXenes for tumor‐specific phototherapy, this surface nanopore engineering on Ti_3_C_2_ MXene integrates several unique features for broadening the MXene‐based biomedical applications, including sufficient mesopore structure with confined capacity for drug delivery (DOX), enhanced hydrophilicity/dispersity/biocompatibility (PEGylation), and multiple surface chemistry for targeting modification (RGD conjugation) (Figure 3g,k).^80^ In short, such two multifunctionalization strategies are still in infancy in comparison to the extensively studied graphene/graphene oxide (GO) family owing to the synthetic and integrating complexities. It is highly expected that the rapid expansion on synthesis and applications of 2D MXenes will promote the emergence of diverse 2D MXene‐based multifunctional nanoplatforms for biomedical applications.

## 3 Therapeutic Applications of MXenes

The unique transformable 2D in‐layer nanostructure and controllable chemical compositions endow 2D MXenes with versatile properties in benefiting biomedical applications. To date, these 2D multifunctional MXenes and their composites have been developed for theranostic applications including typical phototherapy of photothermal therapy (PTT), photothermal/photodynamic/chemo synergistic therapy, diagnostic imaging, antimicrobial, and biosensing. In this section, we introduce various therapeutic applications of diverse MXenes 2D materials.

### 3.1 Photothermal Therapy

**Figure 4:**
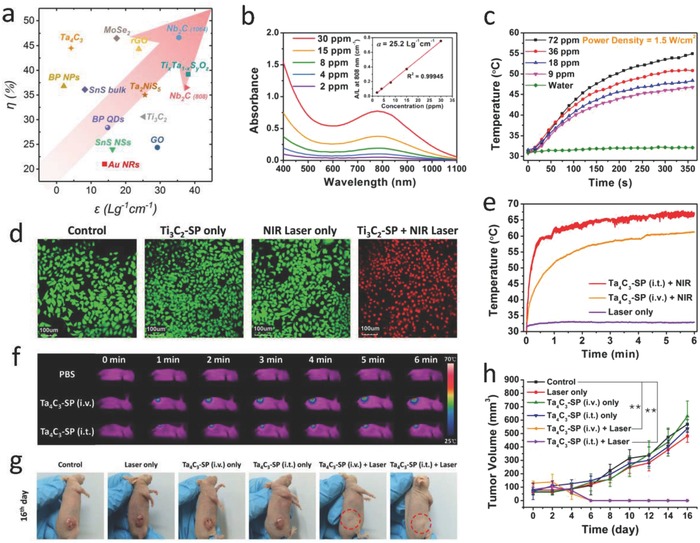
MXene‐based photothermal therapy. a) The photothermal performance parameters, including mass extinction coefficient (ε) and photothermal conversion efficiency (η), of various 2D inorganic nanomaterials in the literatures. Each symbol indicates a set of material category. b) Absorbance spectra of Ti_3_C_2_ nanosheets dispersed in water at varied concentrations (30, 15, 8, 4, and 2 µg mL^−1^). Inset is the mass extinction coefficient (ε) of Ti_3_C_2_ MXene. c) Photothermal‐heating curves of Ti_3_C_2_ nanosheet‐dispersed aqueous suspension at varied concentrations (72, 36, 18, and 9 µg mL^−1^) by using an 808 nm irradiation (1.5 W cm^−2^). d) Confocal laser scanning microscopy (CLSM) images of Ti_3_C_2_‐SP‐induced photothermal ablation after various treatments. Reproduced with permission.^45^ Copyright 2017, American Chemical Society. e) Temperature elevations and f) the corresponding IR thermal images at the tumor sites of 4T1 tumor‐bearing mice in groups of different treatments. g) Photographs of 4T1 tumor‐bearing mice after different treatments. h) Time‐dependent tumor growth curves after different treatments. Reproduced with permission.^47^ Copyright 2017, Wiley‐VCH.

Light is an external stimulus, furnished with extensive profits in efficacy for cancer phototherapeutic modality. Typically, PTT employs photothermal agents accumulated within tumors as internal energy absorbers to convert near‐infrared (NIR) light energy into heat, producing necrosis and/or apoptosis of cancer cells.^81^, ^82^ NIR laser‐based PTT for cancer eradication has attracted considerable interest, which promotes local hyperthermia to ablate tumor tissues with poorly vascularized tumor microenvironment (TME). The exploitation of NIR light as an outer and remote trigger brings high spatiotemporal regulation of local heating effect while minimizes adverse side effects.^83^, ^84^ Photothermal performance of a phototherapeutic agent used for photothermal conversion are determined by two fundamental parameters: the extinction coefficient (ε) and photothermal conversion efficiency (η). The extinction coefficient reveals the light‐absorption capability while the photothermal conversion efficiency indicates the performance of the agent in converting the light into heat.^49^ The photothermal performance parameters of the classic inorganic photothermal agents and 2D inorganic photothermal agents have been summarized in **Figure** **4**a, which indicates the remarkable advantages of emerging 2D MXenes used as photothermal agents, even superior to most of the inorganic photothermal agents in the literatures. Very recently, we and other research groups have achieved a series of breakthroughs on exploiting ultrathin MXenes nanosheets for PTT, including effective photothermal ablation of tumors in a mouse model (e.g., Ti_3_C_2_ MXene),^45^, ^48^ theranostic nanoplatform combining dual‐mode photoacoustic (PA)/CT imaging and in vivo photothermal ablation of mouse tumor xenografts (e.g., Ta_4_C_3_ MXene),^47^ and highly efficient in vivo photothermal tumor eradication and tissue penetration capability in both NIR‐I and NIR‐II biowindows (e.g., Nb_2_C MXene).^49^

As a paradigm of biocompatible 2D MXenes, monolayer or few‐layer Ti_3_C_2_ nanosheets (MXenes) were first fabricated based on a liquid exfoliation method of MAX phase Ti_3_AlC_2_ combining stepwise HF etching and TPAOH intercalation procedures.^45^ Especially, the ultrathin Ti_3_C_2_ nanosheets exhibit an especially high extinction coefficient (ε) of 25.2 L g^−1^ cm^−1^, which is remarkably higher than that of GO nanosheets (3.6 L g^−1^ cm^−1^),^85^ and also notably larger than that of traditional Au nanorods (13.9 L g^−1^ cm^−1^),^86^ indicating a favorable NIR laser‐absorption property of Ti_3_C_2_ nanosheets (Figure 4b). The photothermal conversion efficiency (η) was calculated to be 30.6%, higher than that of Au nanorods (21%),^87^ and Cu_9_S_5_ nanocrystals (25.7%).^88^ Typically, at a low Ti_3_C_2_ concentration (72 µg mL^−1^) of suspension solution, the temperature reached 57 °C upon NIR irradiation in 6 min. In contrast, the control group shows nearly no temperature elevation, implying that the employing of Ti_3_C_2_ nanosheets can rapidly convert NIR light into hyperthermia (Figure 4c). Moreover, the in vitro cell apoptosis after photothermal ablation was further confirmed by confocal laser scanning microscopy (CLSM) imaging, which showed that the majority of 4T1 cells were killed by the photothermal ablation after treatment with Ti_3_C_2_‐SP under NIR laser irradiation, exhibiting the noticeable in vitro photothermal effect of the Ti_3_C_2_‐SP nanosheets in promoting cancer cell ablation (Figure 4d). Furthermore, Ti_3_C_2_ nanosheets were revealed to be a highly effective PTT agent for tumor hyperthermia, which enables excellent NIR light‐induced tumor ablations without recurrence by either intravenous injection of Ti_3_C_2_‐SP (20 mg kg^−1^) or localized intratumoral injection of PLGA/Ti_3_C_2_ phase‐changeable implant (2 mg kg^−1^).^45^

Though Ti_3_C_2_ nanosheets, as the first reported MXene‐based photothermal agent, exhibited a considerable in vivo PTT efficacy, the relative low photothermal conversion efficiency would largely hinder their further broad applications. Considering the flexible element candidates as the in‐layer component of MXenes, the species of early translation metal, M element, could be selected specifically to enhance the element‐based multifunctionalities of MXenes. The 2D Ta_4_C_3_ MXene, a rarely studied type of MXenes possessing biocompatible Ta element, with ultrathin sheet‐like morphology and lateral size of ≈100 nm, has been developed for PTT of tumors.^47^ The absorption spectra acquired on Ta_4_C_3_ MXenes feature a board, strong absorption band, which is of similarity to those of classic 2D nanomaterials, such as graphene^81^ and MoS_2_,^89^ providing a desirable photoabsorption property for further photothermal‐transduction process. Attractively, these ultrathin 2D Ta_4_C_3_ nanosheets exhibit an extraordinarily high photothermal conversion efficiency of 44.7%, superior to most of the inorganic photothermal agents (Figure 4a). Subsequently, the fast temperature elevation was verified in animal models. Upon intravenous or intratumoral administration with biocompatible Ta_4_C_3_‐SP, the temperatures of mouse tumor regions rapidly increased from ≈30 to ≈60 °C or from ≈30 to ≈68 °C in 6 min of laser irradiation, respectively (Figure 4e,f). Thus, highly effective in vivo photothermal tumor eradication by Ta_4_C_3_‐SP nanosheets has been successfully demonstrated (Figure 4g,h).

**Figure 5:**
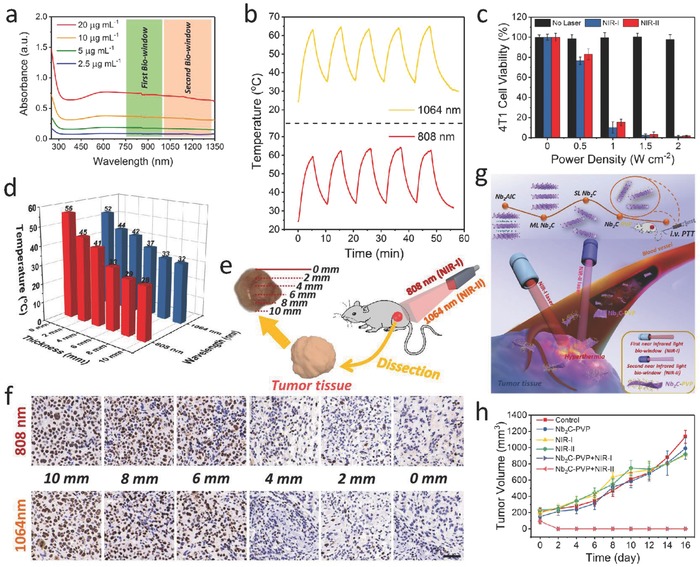
MXene‐based photothermal therapy. a) Absorbance spectra of well‐dispersed aqueous Nb_2_C NSs at varied concentrations. b) Photostability profiles of an aqueous Nb_2_C NSs solution in NIR‐I and NIR‐II biowindows for five laser on/off cycles. c) Relative viabilities of 4T1 cell line after Nb_2_C‐PVP‐induced (40 µg mL^−1^) photothermal eradication at various power densities (0, 0.5, 1.0, 1.5, and 2 W cm^−2^) of laser (*n* = 5, mean ± SD). d) Temperature elevations of Nb_2_C NSs‐dispersed aqueous suspensions upon exposure to tissue‐penetrating NIR‐I and NIR‐II laser via photothermal conversion. e) Schematic diagram of in vivo tumor tissue penetration for photothermal conversion based on NIR‐I and NIR‐II. f) Cancer cellular proliferation at varied depths of tumor tissues by antigen Ki‐67 immunofluorescence staining (scale bar: 50 µm). g) Scheme of synthetic procedure and in vivo photothermal tumor ablation process of 2D biodegradable Nb_2_C (modified with PVP). h) Time‐dependent tumor growth curves after various treatments. Reproduced with permission.^49^ Copyright 2017, American Chemical Society.

Specifically, the majority of previous study focused on the first NIR (NIR‐I) biologic window (noted as biowindow) (750–1000 nm), but the second NIR (NIR‐II) biowindow (1000–1350 nm) has been rarely explored. Compared to the well‐studied NIR‐I biowindow, employing NIR‐II biowindow offers two merits such as desirable depth of NIR laser‐responsive penetration and enhanced maximum permissible exposure (MPE) of biological tissues.^90^ Receiving rapidly increasing attentions, the NIR‐II biowindow, a scientific frontier for biological optical imaging^91^, ^92^ and the phototherapeutics, has aroused great interest in the scientific community of cancer theranostics.^49^, ^93^, ^94^, ^95^ Recently, a novel ultrathin 2D Nb_2_C MXene, as a new phototherapeutic agent, has been exploited in our group for in vivo photothermal ablation of mouse tumor xenograft in both NIR‐I and NIR‐II biowindows. The single‐atomic thickness and lateral‐nanosized Nb_2_C nanosheets feature an extraordinary photothermal performance of broad NIR spectrum with an extraordinarily high photothermal conversion efficiency (**Figure** **5**a,b).^49^ Given that many other types of nanomaterials do show extended absorbance bands in the NIR‐II biowindow, but the absorbances of these photothermal agents, such as MoS_2_,^96^ black phosphorus,^97^ and graphene,^81^, ^85^ evidently decline in the biowindow, which is not favorable for the efficient NIR laser absorption. In contrast, the Nb_2_C nanosheets maintain high‐efficient light absorption in both NIR‐I and NIR‐II biowindow. In contrast, the Nb_2_C nanosheets maintain high light absorption in both NIR‐I and NIR‐II biowindows. In addition, the enhanced tissue penetration depth in NIR‐II window compared to NIR‐I window deserves detailed investigations of its photothermal tumor therapy, which is expected to attract the attention of scientific community for the investigation of photothermal agents with light responsiveness in NIR‐II biowindow. First, the in vitro toxicities of Nb_2_C−PVP to cells showed that with the increase of laser energy, more cells were killed in both NIR‐I and NIR‐II biowindows (Figure 5c). In addition, effective photothermal conversions of the Nb_2_C nanosheets (NSs) were performed in the depth of ex vivo tissue under the penetrated NIR laser irradiation without inducing significant heating of the ex vivo tissue itself. The temperature variations at varied depth intervals of the ex vivo tissues upon NIR‐I and NIR‐II laser radiations indicate that NIR‐II biowindow promotes the photothermal heating with diminished attenuation, in contrast to that of NIR‐I biowindow (Figure 5d). For further investigating the effective tissue penetration of photothermal tumor ablation in NIR‐I and NIR‐II biowindows in vivo, Nb_2_C‐PVP‐treated tumor‐bearing mice were exposed to 808 or 1064 nm laser radiations (Figure 5e). It could be found that both NIR‐I and NIR‐II biowindows allowed the effective photothermal tumor eradication deep to around 4 mm inside subcutaneous tumor xenograft in nude mice (Figure 5f). Furthermore, these surface‐engineered Nb_2_C NSs exhibit highly efficient in vivo photothermal eradication of tumor xenografts in both NIR‐I and NIR‐II biowindows (Figure 5g,h). The aforementioned ex vivo and in vivo evaluations suggest the great promise of using Nb_2_C‐PVP NSs for deep‐tissue PTT in both NIR‐I and NIR‐II biowindows.

### 3.2 Synergistic Therapy

**Figure 6:**
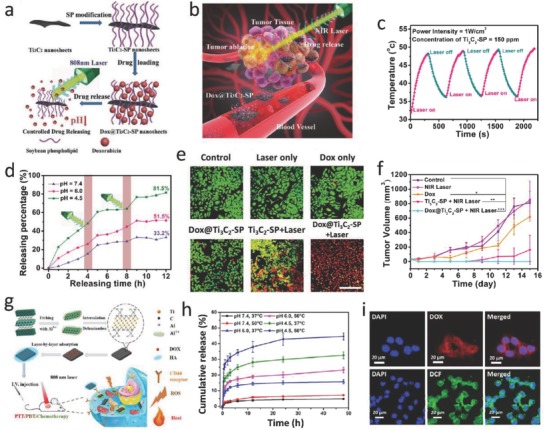
MXene‐based synergistic multitherapies. a) Schematic illustration of surface modification of Ti_3_C_2_ MXene, further surface engineering of drug loading, and stimuli‐triggered drug releasing under inner and external triggers. b) In vivo process of Ti_3_C_2_ MXene‐based drug delivery system (DDS) for synergistic photo‐chemotherapy of tumor. c) The DOX‐releasing curves from DOX@Ti_3_C_2_ composite nanosheets in buffer with varied pHs of 4.5, 6.0, and 7.4. d) The DOX‐releasing curves with 808 nm laser irradiation on or off at varied pH values (4.5, 6.0, and 7.4). e) CLSM images of cancer cells after various treatments, including control, laser, DOX, DOX@Ti_3_C_2_‐SP, Ti_3_C_2_‐SP + laser, and DOX@ Ti_3_C_2_‐SP + laser groups. Scale bar: 50 µm. f) Time‐dependent tumor growth profiles after different treatments. Reproduced with permission.^104^ Copyright 2018, Wiley‐VCH. g) Schematic diagram of the construction of Ti_3_C_2_‐based nanosystem and photothermal/photodynamic/chemo synergistic therapy of tumor. h) Accumulative drug release curves with or without NIR laser irradiation at pH 4.5, 6.0, and 7.4. i) CLSM images of HCT‐116 cells treated with Ti_3_C_2_‐DOX (top), and DCFH‐DA‐stained HCT‐116 cells treated with Ti_3_C_2_‐DOX under NIR laser irradiation for the intracellular ROS detection (bottom). Reproduced with permission.^52^ Copyright 2017, American Chemical Society.

The typical 2D planar topology endows the MXenes with high surface area, allowing easy anchoring of versatile therapeutic agents on the surface of layered structure, similar to delivery feature of typical organic nanoplatforms,^98^ inorganic mesoporous carriers,^99^, ^100^ and other 2D nanoplatforms.^101^, ^102^, ^103^ In consideration of the high photothermal conversion performance of 2D Ti_3_C_2_ MXenes, they were expected to be further employed for highly efficient tumor ablation by synergistic MXene‐assisted photothermal eradication and DOX‐loaded chemotherapy (**Figure** **6**a,b).^104^ The as‐synthesized Ti_3_C_2_ MXenes show desirable photothermal stability during the four‐cycle processes of heating and cooling, which potentially guaranteed the continuous photothermal ablation of tumor and the hyperthermia‐enhanced drug delivery process (Figure 6c). Especially, the Ti_3_C_2_ MXenes as drug delivery nanoplatform not only possess the high capability of drug loading as 211.8%, but also enables both pH‐responsive and NIR laser‐triggered drug releasing (Figure 6d). CLSM was further employed to demonstrate that the integration of Ti_3_C_2_‐promoted PTT with chemotherapy caused the death for almost complete 4T1 cells (Figure 6e). Notably, the integration of Ti_3_C_2_‐assisted PTT with chemotherapy has achieved the complete tumor eradication without reoccurrence in therapeutic period on 4T1 tumor‐bearing mice model, exhibiting the desirable synergistic outcome of PTT and chemotherapy (Figure 6f).

Photodynamic therapy (PDT) features the merits of minimal invasiveness and spatiotemporal specificity for oncologic intervention. Critically, the excited photosensitizer (PS) will transfer its excited‐state energy to molecular oxygen species for generating reactive oxygen species (ROS), inducing tumor‐cell death upon the key cellular substances being oxidized.^105^, ^106^ Upon integrating the cargo delivery with phototherapeutic modality such as PTT and PDT, a synergistic therapy combining chemotherapy with phototherapy could be established. MXene‐based nanotherapeutic agent is highly expected to introduce the concurrent PTT/PDT/chemotherapy‐based multitherapeutic modality to realize the high‐efficient synergistic therapeutics. As a paradigm, a new MXene‐based nanoplatform has been constructed via layer‐by‐layer surface modification of Ti_3_C_2_ nanosheets with doxorubicin (DOX) as the chemotherapy drug and hyaluronic acid (HA) as tumor‐targeting agent (Figure 6g).^52^ This Ti_3_C_2_ nanosheet‐based multifunctional nanoplatform (noted as Ti_3_C_2_‐DOX) shows an as high as 84.2% drug loading capacity. As expected, the Ti_3_C_2_‐DOX exhibits an efficient pH‐responsive drug‐releasing behavior (in mild acidity), benefiting from the effective protection by the HA shell in neutral environment. Meanwhile, the introduction of NIR laser‐induced photothermal effect of Ti_3_C_2_ (temperature up to 50 °C) could enhance the drug‐delivery efficiency in acid TME (Figure 6h). After the intracellular endocytosis of Ti_3_C_2_‐DOX, a NIR laser of 808 nm was irradiated to induce the photothermal effect for hyperthermia and photodynamic process for ROS generation (Figure 6i). It is highly attractive that the effective synergistic therapeutic outcome has been achieved by the facile therapeutic drug loading on the 2D MXenes beyond the tedious material structure design, providing a paradigm of synergistic tumor therapy based on other 2D nanosystems.

**Figure 7:**
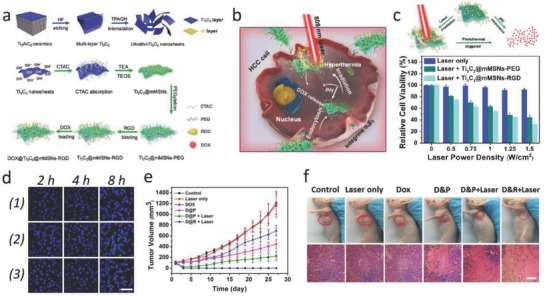
MXene‐based synergistic multitherapies. a) Schematic diagram for the fabrication of ultrathin Ti_3_C_2_ nanosheets and the synthetic procedure for Ti_3_C_2_@mMSNs‐RGD. b) Scheme for synergistic multitherapies on HCC cell line as assisted by DOX‐loaded mMSNs@Ti_3_C_2_‐RGD. c) Schematic representation of pH/photothermal‐responsive drug release from DOX‐loaded Ti_3_C_2_@mMSNs‐RGD (top), and relative viabilities of SMMC‐7721 cells under NIR irradiation of different power densities (0, 0.5, 0.75, 1, 1.25, and 1.5 W cm^−2^) (bottom). d) CLSM images of SMMC‐7721 cell line coincubated with FITC‐labeled mMSNs@Ti_3_C_2_ for a varied incubation times (2, 4, and 8 h). Scale bar: 50 µm. e) Time‐dependent tumor growth curves after various treatments. f) Photographs of SMMC‐7721 tumor‐bearing mice and its tumor regions in 28 d after various treatments. H&E staining for pathological changes in tumor tissues from each group. Scale bar: 100 µm. Reproduced with permission.^80^ Copyright 2018, Wiley‐VCH.

Active‐targeting technique is a promising pathway to enhance the accumulation of drug carriers or therapeutic agents toward disease region. A nanopore engineering has been performed on the surface of MXenes, as‐synthesized RGD‐targeted Ti_3_C_2_@mMSNs‐RGD, endowing synergistic multitherapies of MXenes with well‐defined mesoporous nanostructure for drug delivery and release, tumor‐specificity for active‐targeting response, and inherent photothermal conversion performance for PTT (**Figure** **7**a,b).^80^ The RGD‐targeted photothermal‐ablation efficacy of Ti_3_C_2_@mMSNs‐RGD under NIR laser exhibited that with the increase of laser energy, more hepatocellular carcinoma (HCC) cells incubated with Ti_3_C_2_@mMSNs‐RGD were killed compared to Ti_3_C_2_@mMSNs‐PEG without RGD modification (Figure 7c). Simultaneously, Ti_3_C_2_@mMSNs‐RGD exhibited evident superiority in suppression of HCC cells owing to the active‐targeting capability between RGD peptides anchored on Ti_3_C_2_@mMSNs surface and α_v_β_3_ ligands expressed on HCC cell membranes, facilitating more synergistic therapeutic agents to target the HCC cells via an efficient endocytosis as showed by CLSM (Figure 7d). Systematic in vivo evaluations also indicated the high active‐targeting outcome (contributed by RGD) of Ti_3_C_2_@mMSNs‐RGD into tumor, and the synergistic chemotherapy (derived from MSNs) and hyperthermia (contributed by Ti_3_C_2_ MXene) have thoroughly eradicated the tumor without further reoccurrence (Figure 7e,f).

## 4 Contrast‐Enhanced Diagnostic Imaging of MXenes

The versatile physicochemical properties of 2D nanosheets enable great potentials for diagnostic imaging, implying that they could act as the CAs to enhance the diagnostic‐imaging performance.^21^ In comparison to conventional 2D CAs, 2D MXene‐based agents feature quantum size effects for photoluminescence (PL) cellular imaging, intrinsic photothermal performance for PA imaging, element‐enhanced contrast for X‐ray CT imaging, and effective loading of functional CAs for MR imaging.

### 4.1 Fluorescent Imaging

**Figure 8:**
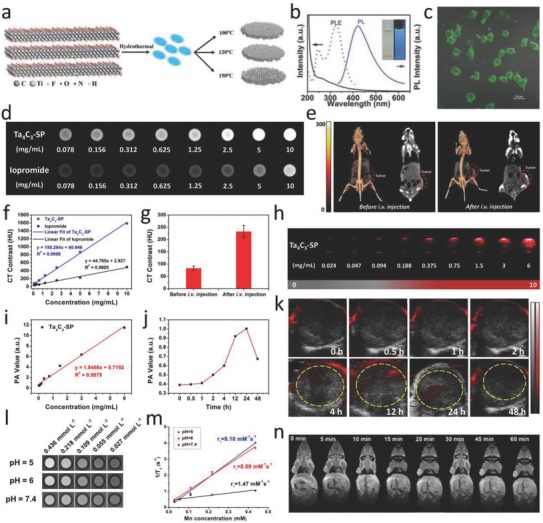
MXene‐based diagnostic imaging modalities. a) Schematic diagram of synthesizing Ti_3_C_2_ MXene QDs (MQDs). b) UV–vis absorbance spectra (solid line), PLE (dashed line), and PL spectra (solid blue line, excitation wavelength of 320 nm) of MQD‐100 in aqueous solutions. c) Merged images of the bright‐field and the confocal images (488 nm) for MQD‐100 incubated with cells. Reproduced with permission.^53^ Copyright 2017, Wiley‐VCH. d) In vitro CT images and f) CT contrasts of Ta_4_C_3_‐SP nanosheet solutions and iopromide solutions at varied concentrations. e) In vivo 3D reconstruction CT images (left) and CT contrast images (right) of mice before and after intravenous administration (10 mg mL^−1^, 200 µL) for 24 h. g) In vivo CT contrasts before and after intravenous administration. h) PA images of Ta_4_C_3_‐SP solutions with varied concentrations. i) In vitro PA values as a function of a series of concentrations. j) In vivo PA value temporal evolution and k) PA images of the tumor locations at varied time intervals postinjection. Reproduced with permission.^47^ Copyright 2017, Wiley‐VCH. l) In vitro T_1_‐weighted MR imaging, and m) the corresponding 1/*T*_1_ versus Mn concentration of MnO*_x_*/Ti_3_C_2_‐SP nanosheets in buffer solution at different pH values after soaking for 3 h. n) *T*_1_‐weighted imaging of tumor‐bearing mice after postinjection of MnO*_x_*/Ti_3_C_2_‐SP at varied time intervals. Reproduced with permission.^51^ Copyright 2017, American Chemical Society.

Compared to traditional organic fluorescein, inorganic 2D nanomaterials and their corresponding quantum dots (QDs) have shown intriguing fluorescent property for bioimaging such as tunable wavelength, high photostability, and desirable quantum yields. For instance, the graphene QDs, as the first developed 2D metal‐free fluorescent nanomaterial, have found their potential utilization for efficient fluorescent imaging.^107^ The ultrathin liquid‐exfoliated g‐C_3_N_4_ and corresponding g‐C_3_N_4_ QDs also exhibited high photoluminescence quantum yield for intracellular bioimaging.^108^, ^109^ The luminescent Ti_3_C_2_ MXene‐based quantum dots (noted as Ti_3_C_2_ MQDs) with hydrophilic nature and monolayer structure were synthesized by a facile hydrothermal process. The physiochemical property and PL principle of the as‐prepared Ti_3_C_2_ MQDs under different synthetic conditions have been extensively studied (**Figure** **8**a).^53^ These Ti_3_C_2_ MQDs fabricated at 100 °C (MQDs‐100) feature excitation‐dependent PL behavior of a high quantum yield up to 9.9%, which possibly derives from the strong quantum confinement effect (Figure 8b). As indicated in the merged CLSM image, the MQDs were easily taken up via the endocytosis pathway (Figure 8c). The application of MQDs as multicolor cellular‐imaging reagents was demonstrated via labeling RAW264.7 cell line, which exhibit the high potential of MXene‐based QDs for applications in areas of optics, biomedicine, and cellular imaging. As another example, Geng and co‐workers reported a strategy for the fabrication of ultrasmall MXenes by an intralayer cutting and delamination approach under a mild condition with aqueous tetramethylammonium hydroxide (TMAOH).^110^ The as‐obtained ultrasmall Ti_3_C_2_ MXenes feature strong optical absorption and excitation‐dependent emission. Importantly, such a synthetic approach could also be extended to fabricating ultrasmall dots of other members in MXene family, such as Ti_2_C and Nb_2_C. More recently, Wang and co‐workers investigated the luminescent property from solvothermal‐treated Ti_3_C_2_T*_x_* MXene in dimethylformamide (DMF), and demonstrated their application in cellular imaging.^111^

### 4.2 Computed Tomography Imaging

X‐ray CT has been widely used for medical imaging modality because of its 3D tomography of the anatomic structure based on the differential X‐ray absorptions between the lesions and tissues.^112^ Nanomaterials containing high atomic number (*Z*) elements have frequently been explored as CT CAs. WS_2_‐PEG as the CA for CT imaging was well developed owing to the higher *Z* of W (*Z* = 74) superior to the clinical use of I element (*Z* = 53).^113^ In another case, bismuth (*Z* = 83) was expected as a desirable candidate CT CA based on its much higher atomic number (*Z*) than most metal elements and the favorable biocompatibility. Thus, 2D topological Bi_2_Se_3_ nanosheets feature strong X‐ray attenuation and exhibit enhanced CT imaging of tumor tissue in vivo.^114^ In this regard, tantalum (Ta)‐based compound, Ta_4_C_3_ MXenes, could no doubt function as a CT CA due to its high atomic number of 73.^47^ The Hounsfield unit (HU) value and corresponding CT images of Ta_4_C_3_‐SP dispersed in Xanthan gum exhibited a strong signal enhancement along with the increased Ta_4_C_3_‐SP contents. The in vitro CT imaging of Ta_4_C_3_‐SP shows enhanced contrast in comparison with the commercial iodine‐based CT CAs (Figure 8d,f). Moreover, the tumor‐bearing mice received intravenous administration of Ta_4_C_3_‐SP for in vivo evaluation. CT images collected in 24 h postinjection indicates distinct tumor contrast enhancement ranging from 83 ± 8.8 to 232.3 ± 25.2 HU (Figure 8e,g). Therefore, these Ta_4_C_3_ MXenes could function as promising CAs for potential CT imaging.

### 4.3 Photoacoustic Imaging

Generally, the efficient photothermal conversion agents could function as CAs for PA imaging. PA imaging, an emerging technology overcoming the high degree of scattering of optical photons in biological tissue by making use of the photoacoustic effect, offers living subjects higher spatial resolution and allows deeper tissues to be imaged compared with most optical imaging techniques. Light absorption by molecules creates a thermally induced pressure jump that launches ultrasonic waves, which are received by acoustic detectors to form images.^115^, ^116^ As many diseases do not exhibit a natural photoacoustic contrast, especially in their early stages, it is necessary to administer a photoacoustic contrast agent. Many conventional 2D nanosystems such as graphene, MoO*_x_*, and WS_2_ have been developed to offer strong contrasts under PA imaging.^96^, ^117^, ^118^ Notably, 2D MXenes are also expected to play this role. Owing to the desirable photothermal conversion efficiency of Ta_4_C_3_ MXene in the NIR biowindow, PA imaging was investigated by using the Ta_4_C_3_‐SP nanosheets as CAs.^47^ In detail, in vitro PA images and the corresponding values of Ta_4_C_3_‐SP at varied concentrations obviously indicate their contrast‐enhancement functions (Figure 8h,i). Then, tumor‐bearing mice were intravenously injected with Ta_4_C_3_‐SP, and the corresponding in vivo PA images were collected at varied time intervals (Figure 8j,k). In comparison with the pretreatment image, the PA signal of the tumor region exhibits a time‐dependent lightening process featuring intensity increase from 0.39 to 1.0 a.u., a highly significant value in about 24 h postinjection possibly resulting from the passive accumulation of Ta_4_C_3_ MXene nanosheets via the enhanced permeability and retention (EPR) effect.^119^ So far, we and other groups have reported on various MXene and their compounds for in intro and in vivo PA imaging, including Ti_3_C_2_, Nb_2_C, Ta_4_C_3_, and MnO*_x_*/Ti_3_C_2_, under an NIR light excitation.^46^, ^47^, ^49^, ^51^

### 4.4 Magnetic Resonance Imaging (MRI)

Due to the high spatial resolution, excellent contrast difference of 3D soft tissues, and noninvasive feature, MRI has been extensively applied for clinically diagnostic imaging.^120^, ^121^ Integration of functional components with ultrathin 2D nanosheets paves a new way for multifunctionalization of 2D nanosystems, which largely broadens their multiple applications in diagnostic‐imaging fields. For example, the integration of MnO*_x_* with Ti_3_C_2_ profits the Mn‐based MRI, one of the clinic‐used effective imaging modalities because of its advanced spatial resolution and desirable tissue contrast,^10^ which has been investigated for diagnostic tumor MR imaging. The MnO*_x_* component in MnO*_x_*/Ti_3_C_2_ features the specific pH‐responsive *T*_1_‐weighted MRI capability upon arriving at the acidic TME. An evident concentration‐dependent enhancement was observed in *T*_1_‐weighted MRI, and an acidic‐induced positive MRI signal intensification was clearly revealed (Figure 8l,m).^51^ Further in vivo *T*_1_‐weighted MRI evaluation of MnO*_x_*/Ti_3_C_2_‐SP nanosheets was conducted on tumor‐bearing mice. A significant enhancing effect of MRI contrasts was demonstrated in tumor sites, owing to the efficient accumulation of the MnO*_x_*/Ti_3_C_2_‐SP nanosheets through the EPR effect and the Mn ion release in the mild acidic TME (Figure 8n). In another case, we developed MnO*_x_*/Ta_4_C_3_ composite nanosheets acted as the high‐performance contrast agents for TME‐responsive *T*_1_‐weighted MR imaging, which also derived from the MnO*_x_* component.^46^

## 5 Biosensing

Beyond the versatile applications of 2D nanomaterials for therapeutics and diagnostic imaging, they could also be employed as novel biosensing systems for the detection of biomacromolecules and bio‐effects. Monolayer MoS_2_ nanosheets have been used as a biosensor for DNA detection based on their strong fluorescence‐quenching effect.^122^ In another example, based on 2D g‐C_3_N_4_ nanosheet, a DNA biosensor was designed by utilizing the affinity changes of g‐C_3_N_4_ to DNA probes upon their targeting the analyte and the related positron emission tomography (PET)‐based fluorescence‐quenching effect.^123^ In comparison with traditional nanoparticle‐based biosensors, 2D nanostructures feature two prominent merits in biosensing. One is the high surface‐to‐volume ratio of 2D layered structure facilitating large‐area immobilization of sensing targets, and the other is the fascinating performances such as light‐absorption capability and fast electron transfer fluorescence‐quenching effect based on the unique physicochemical property of 2D nanosheets.^29^ Indeed, there is a continuous demand for the development of highly sensitive, selective, efficient, and cost‐effective biosensing platforms. On this ground, MXenes, with a hydrophilic surface nature, metallic conductivity property, and planar atomic structure, could be expected as a promising candidate in manufacturing biologically compatible field‐effect transistors (FET) for fast, direct, and label‐free analysis of biological events.^54^

**Figure 9:**
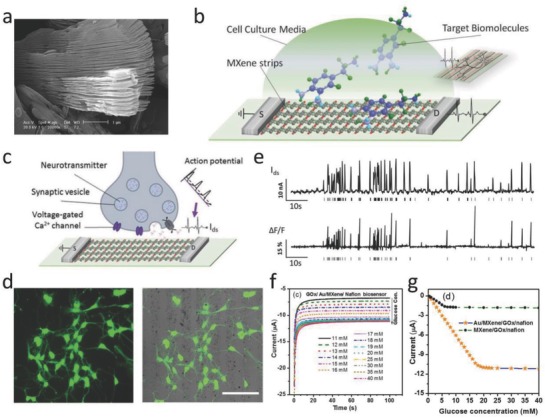
MXene‐based applications in biosensing. a) SEM image of multilayered Ti_3_C_2_ MXene. b) Device schematics for biosensing based on Ti_3_C_2_ MXene field‐effect transistor (FET). c) Schematics of real‐time recording for neuronal‐spiking activities by employing MXene‐FET device. d) Confocal image of neurons immunofluorescence staining for βIII‐tubulin (left). Merged image of the bright‐field and fluorescence channels (scale bar: 100 µm). e) The derivation of spiking activities from primary neurons by utilizing MXene‐FET device via recording the current and fluorescence changes. Reproduced with permission.^57^ Copyright 2017, Wiley‐VCH. f) Amperometric current–time (*i*–*t*) curves for GO*_x_*/Au/MXene/Nafion/GCE biosensor under a constant voltage of −0.402 V. g) Steady‐state calibration curves for contrast recordings between GO*_x_*/MXene/Nafion/GCE and GO*_x_*/Au/MXene/Nafion/GCE biosensors. Reproduced with permission.^55^ Copyright 2016, Nature Publishing Group.

Recently, a highly sensitive device based on MXene‐FET‐based biosensor with high sensitivity was developed for performing label‐free detection of dopamine, and monitoring spiking activity in primary hippocampal neurons.^57^ The as‐synthesized MXene stripes of multilayered Ti_3_C_2_ offer an opportunity for the active surface to be in contact with small biomolecules, achieving the conductivity signal changing in turn. The high sensitivity of response‐to‐signal perturbation of electrochemistry enables MXenes to be potential candidates for biosensing alternatives (**Figure** **9**a,b). The firing of action potentials results in the release of neurotransmitters, inducing the electric signal changing by the subsequent binding between neurotransmitters and MXene surface (Figure 9c). The neurons display normal, elaborated neurites on the surface of MXene substrates, indicating that surface‐modified MXenes have a high biocompatibility with neuron cells (Figure 9d). The spike curve of action potentials originating from electrical signals and optical recording of fluorescence changes exhibit a high correlation coefficient of 0.82, which endows MXene‐FET device with high temporal resolution (Figure 9e). These results clearly demonstrate the strong correlation between electrical signals and the release of action potentials, and confirm that the MXene‐based biosensors are promising alternatives for real‐time probing of neural activities. In addition, MXene composite‐based biosensor also exhibits high sensitivity, broad sensitive range, and excellent stability. For instance, the Au/MXene nanocomposite platform was developed for the detection of sensitive enzymatic glucose.^55^ A general trend of increasing current at increased glucose concentration has been observed in the measurement of GO*_x_*/Au/MXene/Nafion/GCE biosensors (Figure 9f), which exhibits a linear amperometric response in the glucose concentration range from 0.1 to 18 × 10^−3^ m with a relatively high sensitivity of 4.2 µA mM^−1^ cm^−2^ and a detection limit of 5.9 × 10^−6^ m (Figure 9g).

## 6 Antimicrobial Activity

Various 2D nanomaterials have been explored for their potential antibacterial activities following different strategies. For instance, after the addition of Zn‐Ti layered double hydroxides (LDHs) to bacteria suspension under visible light, the growth of microbial species such as *S. cerevisiae, S. aureus*, or *Escherichia coli* was strongly inhibited because of the LDH size effect and generation of ROS by Ti^3+^ under visible light.^124^ The antimicrobial behaviors of chemically exfoliated MoS_2_ (ce‐MoS_2_) were attributable to both membrane and oxidation stress, which were investigated by addition of a ce‐MoS_2_ suspension to the bacterial culture.^125^

**Figure 10:**
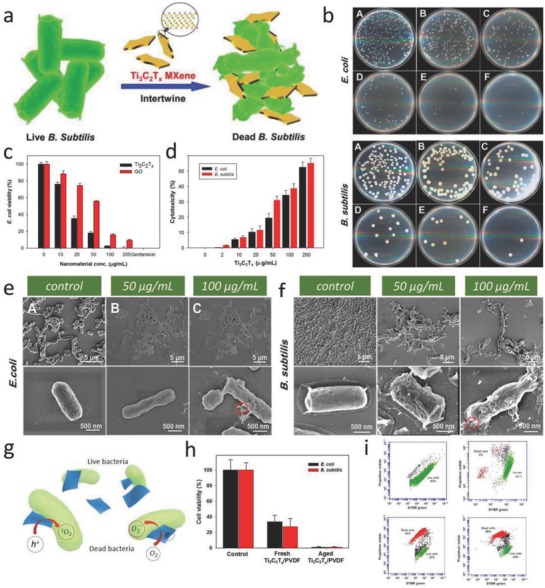
MXene‐based applications in antimicrobials. a) Schematic illustration of antibacterial activity of Ti_3_C_2_T*_x_* MXene. b) Concentration‐dependent antibacterial activities of the Ti_3_C_2_T*_x_* in aqueous suspensions: Images of agar plates onto which *E. coli* (top) and *B. subtilis* (bottom) bacterial cells were recultivated after treatment for 4 h with varied concentrations. c) Cell viability analysis of *E. coli* treated with Ti_3_C_2_T*_x_* and graphene oxide (GO) in aqueous suspension. d) Ti_3_C_2_T*_x_* cytotoxicity investigated by LDH‐releasing from the bacterial cells exposure to varied concentrations of Ti_3_C_2_T*_x_*. SEM images of the e) *E. coli* and f) *B. subtilis* treated with 0, 50, and 100 µg mL^−1^ of Ti_3_C_2_T*_x_*, at low and high magnification, respectively. Reproduced with permission.^58^ Copyright 2016, American Chemical Society. g) The scheme of antimicrobial mechanism for Ti_3_C_2_ MXenes. h) Cell viability studies of *E*. *coli* and *B*. *subtilis* cultivated on fresh and aged PVDF‐supported Ti_3_C_2_ membranes. i) Flow cytometry analyses of *E*. *coli* and *B*. *subtilis* bacterial cells exposed to PVDF and Ti_3_C_2_ MXene membranes. Reproduced with permission.^59^ Copyright 2017, Nature Publishing Group.

In this respect, to extensively exploit the environmental and health impacts of these emerging 2D MXenes, the antimicrobial activities of typical single‐ and few‐layer Ti_3_C_2_ MXenes toward two bacterial models, *E. coli* and *Bacillus subtilis*, were investigated and compared with other carbon‐based nanomaterials (**Figure** **10**a).^58^ In detail, the antibacterial activities of Ti_3_C_2_ MXenes were evaluated against *E. coli* and *B. subtilis* by employing bacterial growth curves and colonies growth assays (Figure 10b). Ti_3_C_2_ exhibits a higher antimicrobial activity toward both *E. coli* and *B. subtilis* in comparison with GO, a well‐studied antimicrobial agent. The concentration‐dependent antibacterial activity was revealed, and Ti_3_C_2_ MXenes incubated with both bacterial cells within 4 h in a colloidal solution (200 µg mL^−1^) led to >98% viability losses of the bacterial cells (Figure 10c). Furthermore, LDH release assay was employed to quantitatively investigate the extent of cell damage. The cytotoxicity of Ti_3_C_2_ MXenes was measured by LDH release from the bacterial cells exposed to varied concentrations of Ti_3_C_2_T*_x_* nanosheets for 4 h (Figure 10d). LDH release elevated remarkably when bacterial cells were of exposure to 200 µg L^−1^ of Ti_3_C_2_T*_x_* solution, exhibiting cytotoxicity of 38.41 and 55.24% for *E. coli* and *B. subtilis*, respectively. Such dose‐dependent cytotoxicity indicates that both the walls and the inner contents of the bacterial were damaged, demonstrating that membrane disruption perhaps function as a major cell suppressive mechanism. In order to visually characterize the antibacterial effect of Ti_3_C_2_T*_x_* MXene, changes of morphology and membrane integrity of *E. coli* and *B. subtilis* cells due to the interaction with Ti_3_C_2_T*_x_* were further evaluated by scanning electron microscopy (SEM) (Figure 10e,f). With elevating concentration of Ti_3_C_2_T*_x_* comes more damage of bacteria, such significant change in the cell morphology/structure could be contributed by detachment of the cytoplasmic membrane from the bacterial cell wall, which was well‐matched with the outcome of LDH release assay.

As another example, the antibacterial property of Ti_3_C_2_T*_x_*‐modified membrane was investigated against *E. coli* and *B. subtilis* by bacterial growth on the membrane surface.^59^ The antibacterial rate of fresh Ti_3_C_2_T*_x_* MXene membranes reaches more than 67% against *E. coli* and 73% against *B. subtilis* as compared with that of control samples, while aged Ti_3_C_2_T*_x_* membrane exhibited more than 99% growth suppression of both bacteria species (Figure 10h). Flow cytometry depicted almost 70% of dead and compromised cells after 24 h exposure of both bacterial colonies in the surface of PVDF and Ti_3_C_2_T*_x_* (Figure 10i). The demonstrated antibacterial activity of MXene‐based membranes against common bacteria species facilitates their potential application as anti‐biofouling membrane in water and wastewater treatment technology. The possible antimicrobial mechanism of Ti_3_C_2_ MXenes has been proposed as follows: First, sharp edges of Ti_3_C_2_ MXenes enable their effective adsorbing on the surface of microorganisms. Second, exposure of microorganisms to the sharp edges of the MXenes might induce membrane damage. Third, Ti_3_C_2_ MXenes are likely to react with biomolecules in the cell wall and cytoplasm of microorganisms, breaking the cell microstructure and triggering the death of microorganism (Figure 10g).^58^ Therefore, MXenes should be introduced as a new family of 2D antimicrobial agents for their potential use in areas of health and environmental protection.

## 7 Biosafety/Cytotoxicity Evaluations of MXenes

Traditional organic materials with high biocompatibility/biodegradation have been explored for biomedical applications, unfortunately their poor chemical/thermal stabilities and single functionality are the drawbacks impeding their clinical development.^126^ In comparison, inorganic 2D MXene‐based nanomaterials show relatively high clinical translation potential, which derives from their inherent characteristics such as facile functionalization, tunable morphology/structure, desirable biocompatibility, specific biodegradation, multifunctionality, and relatively high physiological stability. These specific features are usually difficult to achieve on most organic entities. Although MXenes feature unique properties in biomedical practices, the biosafety of emerging 2D MXenes family determines their potentials in future clinical applications.

**Figure 11:**
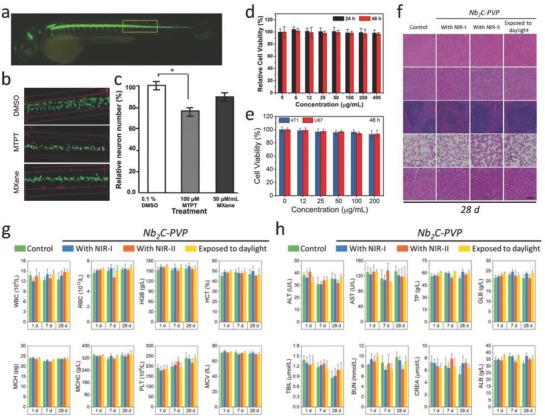
Ecotoxicological assessments, biocompatibility, and biosafety evaluations of MXenes. a) CLSM imaging of an entire embryo. b) Spinal cord adjacent to the somite 14–17 territory of the various treatments toward representative embryos. c) Average of the relative neuronal number of as‐treated embryos (*n* = 14, *P < 0.05). Reproduced with permission.^129^ Copyright 2018, Royal Society of Chemistry. d) Relative viabilities of 4T1 cell line after being incubated with varied concentrations of Ta_4_C_3_‐SP nanosheets. Reproduced with permission.^47^ Copyright 2018, Wiley‐VCH. e) Relative viabilities of 4T1 and U87 cells after being incubated with Nb_2_C‐PVP nanosheets of varied concentrations. f) Histological data (H&E stained) collected from the major organs (heart, liver, spleen, lung, and kidney) of the Nb_2_C‐PVP‐treated mice in 28 d postinjection under different conditions (control, exposure to NIR‐I, NIR‐II, and daylight). Scale bars: 100 µm. g) Hematological parameters and h) biochemical blood indexes of the Nb_2_C‐PVP‐treated mice in 1, 7, and 28 d postinjection under different treatments (control, exposure to daylight, NIR‐I, and NIR‐II). Reproduced with permission.^49^ Copyright 2017, American Chemical Society.

The toxicity/biocompatibility evaluations of various types of 2D nanomaterials have been under progress, the impacts of biomedicine derived from crucial parameters of 2D nanomaterials, such as solubility, dispersibility, long‐term toxicity, and biodegradation, are still unknown.^127^, ^128^ In a recent work, the potential ecotoxicological assessments of MXene‐based nanomaterials in vivo on an aquatic biota were conducted on a zebrafish embryo model, which were expected to be able to indirectly reveal the possible adverse toxicological effect on human health. The neurotoxicity and locomotion assessments showed that the neuron number in the spinal cord area of Ti_3_C_2_T*_x_*‐treated zebrafish embryos was similar to that of dimethyl sulfoxide (DMSO) negative control (**Figure** **11**a), suggesting that at the no observed effect concentration (NOEC) (50 µg mL^−1^), Ti_3_C_2_T*_x_* MXene had no adverse toxicological effect on the neuron and muscle activity of the zebrafish embryo model (Figure 11b,c). Essentially, as the LC_50_ of Ti_3_C_2_T*_x_* exceeds to 100 µg mL^−1^, Ti_3_C_2_T*_x_* MXene could be classified in the “practically nontoxic” group in accordance with the Acute Toxicity Rating Scale by the Fish and Wildlife Service (FWS).

In addition, there are promising progresses demonstrating their potentially high biocompatibility in biomedicine. It has been found that both MXenes and the MXene‐based composites introduce low cytotoxicities toward cells. For example, the Ti_3_C_2_‐SP nanosheets show negligible effect on the survival of 4T1 cell line for 48 h coincubation, even at a high concentration of 400 µg mL^−1^.^45^ The in vitro toxicities of Ta_4_C_3_‐SP upon cells were tested by a standard Cell Counting Kit‐8 (CCK‐8) assay. Ta_4_C_3_‐SP also shows negligible impact on the survival of 4T1 cell line, even at the concentration up to 400 µg mL^−1^ (Figure 11d). Moreover, two cancer cell lines of glioma U87 cancer cells and breast 4T1 cancer cells were incubated with Nb_2_C‐PVP, indicating no significant suppression effect on both U87 cells and 4T1 cells, even at the concentration up to 200 µg mL^−1^ (Figure 11e).^49^ In the case of MXene‐based composite nanosheets (MnO*_x_*/Ti_3_C_2_‐SP), negligible cytotoxicity was found even at a high concentration of 160 µg mL^−1^ after coincubation for 48 h, indicating the relatively high biocompatibility of MnO*_x_*/Ti_3_C_2_‐SP composite nanosheets.^51^

The further in vivo potential biosafety and biocompatibility of surface‐modified MXene nanosheets against mice were comprehensively evaluated. For instance, healthy Kunming mice with three dosages of surface‐modified MXenes (Nb_2_C‐PVP dosage of 20 mg kg^−1^) were assessed in 1, 7, and 28 d.^47^ It was found that negligible abnormal behavior of mice was recorded between three treated groups (exposure to NIR‐I, NIR‐II, and daylight) and the control group, and no abnormality was observed on body weights of mice within three observation period. The hematoxylin and eosin (H&E) staining assays of major organs (heart, liver, spleen, lung, and kidney) after a 28 d feeding exhibit no obvious acute, chronic pathological toxicity, and side effects compared the control group with the treated groups (Figure 11f). The blood tests, including hematological indexes and biochemistry parameters, were in the normal range (Figure 11g,h). These results indicate that Nb_2_C‐PVP is potentially biocompatible for further in vivo therapeutic modalities of tumor theranostics. The same assessments were performed on Ti_3_C_2_ and Ta_4_C_3_ MXenes,^45^, ^49^ which feature negligible cytotoxicity and satisfactory in vivo biocompatibility. However, in light of the current results, further investigation and optimization of in vitro and in vivo toxicity assessment such as genotoxicity^127^, ^128^ or reproductive toxicity^130^ are highly needed to employ the full potential of MXene‐based theranostic nanoplatform for versatile biomedical applications.

## 8 Conclusions and Perspectives

This review highlights the current research progress in the design and production of pristine and functionalized MXenes and their composites, with particular emphasis on their biological and biomedical applications, including therapeutic modality, diagnostic imaging, biosensing, antimicrobial, and biosafety evaluation (**Table** **1**). It is noted that the development of 2D MXenes in nanomedicine is still in its infancy. The emerging trends in 2D MXenes are the exploitations of their intrinsic physiochemical nature such as controllable in‐plane component, transformable multidimension, and tunable terminating species that are of multifunctionality, programmability, and biocompatibility. These specific properties of 2D MXenes can be achieved and regulated by designing multicomponent or multidimensional nanosystems as well as inventing innovative fabrication strategies.

**Table 1:** Applications of MXenes in nanomedicine

Application	Material	Description	Refs.
PTT	Ti_3_C_2_, Ti_3_C_2_ QDs, Ta_4_C_3_, Nb_2_C, MnO*_x_*/Ti_3_C_2_, MnO*_x_*/Ta_4_C_3_	High photothermal conversion performance (Nb_2_C: η = 36.5% (808 nm) or 46.65% (1064 nm), Ti_3_C_2_: η = 30.6%, and Ta_4_C_3_ = 44.7%), in vitro and in vivo photothermal ablation of tumor.	45, 49, 51, 52, 131
Synergistic PTT/PDT/chemotherapy	Ti_3_C_2_‐DOX	Demonstrating stimuli‐responsive drug‐releasing behavior, superior photothermal conversion efficiency (η = 58.3%), and effective singlet oxygen generation (^1^O_2_), achieving effective cancer cell killing and tumor tissue destruction both in vitro and in vivo.	52
PL imaging	Ti_3_C_2_ QDs	A biocompatible multicolor cellular imaging probe exhibiting excitation‐dependent PL spectra with quantum yields around 10%.	53
MR imaging	MnO*_x_*/Ti_3_C_2_, MnO*_x_*/Ta_4_C_3_	Tumor microenvironment‐responsive (pH‐responsive) contrast agents for *T*_1_‐weighted MR imaging.	46, 51
CT imaging	Ta_4_C_3_, MnO*_x_*/Ta_4_C_3_	High atomic number of Ta component endows Ta_4_C_3_ MXene with contrast‐enhanced computed tomography imaging.	46, 47
PA imaging	MnO*_x_*/Ti_3_C_2_, Ta_4_C_3_, MnO*_x_*/Ta_4_C_3_, Nb_2_C	High photothermal conversion performance of MXenes enables their potential PA imaging.	46, 47, 49, 51
Biosensing	Ti_3_C_2_	MXene‐micropattern‐based FET for probing neural activity, immobilizing hemoglobin (Hb) to fabricate a mediator‐free biosensor, amperometric glucose biosensor.	54, 55, 56, 57
Antimicrobial activity	Ti_3_C_2_, PVDF/Ti_3_C_2_ membrane	Colloidal Ti_3_C_2_T*_x_* solution exhibits antibacterial activity against *E. coli* and *B. subtilis*.	58, 59
Biosafety/cytotoxicity	Ti_3_C_2_, Ta_4_C_3_, Nb_2_C, MnO*_x_*/Ti_3_C_2_, MnO*_x_*/Ta_4_C_3_, Fe_3_O_4_/Ti_3_C_2_, Fe_3_O_4_/Ta_4_C_3_	Ecotoxicological assessments, cytotoxicities, and in vivo potential biosafety and biocompatibility	45, 46, 47, 49, 50, 51, 78, 129, 132

**Figure 12:**
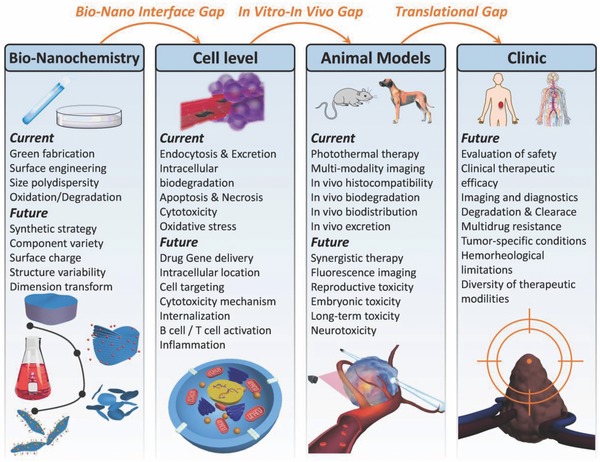
Conclusions and perspectives for 2D MXenes used in nanomedicine. Summary of current research developments and future perspectives of ultrathin 2D MXenes for versatile biomedical applications.

No doubt, numerous unprecedented challenges exist in exploiting 2D MXenes for translation applications such as large‐scale production, precise structure/composition control, targeting surface engineering, and comprehensive and long‐term evaluation on the biocompatibility/biosafety. In addition, the biomedical applications of 2D MXene in other biomedical field should be further explored and promoted, such as stem‐cell engineering, immunological therapy, regenerative medicine, gene therapy, tissue engineering, and possibly other novel cancer treatments (see detailed summary in **Figure** **12**). It is highly expected that the great and multidisciplinary‐joined efforts among biology, chemistry, physics, and engineering will promote mechanistic understanding of MXene‐based systems for nanomedicine and allow currently succeeded paradigms more rapidly and extensively applied in versatile biomedicine.

## Conflict of Interest

The authors declare no conflict of interest.
